# Brunswik’s fundamental principle explained: A diffusion lens model of vicarious functioning

**DOI:** 10.3758/s13423-025-02764-9

**Published:** 2026-02-17

**Authors:** Florian Scholten, Lukas Schumacher, Paul Kelber

**Affiliations:** 1 Department of Psychology, Eberhard Karls Universität Tübingen, Schleichstraße 4, 72076 Tübingen, Germany; 2 University of Basel, Basel, Switzerland

**Keywords:** Lens model, Cue probability learning, Diffusion model, Superstatistics

## Abstract

In Egon Brunswik’s theory of probabilistic functionalism, human prediction is conceptualized as an inductive inference process, in which cues are utilized as a lens to predict the probabilistically associated criterion in the environment. Dynamic cognitive adjustment, driven by the uncertainty of the individual and the substitutability of the environment, is based on vicarious functioning, the principle of learning from the frequency of co-occurrences. However, previous models of vicarious functioning, the multiple-regression lens and the fast-and-frugal lens, fail to explain how the individual reduces uncertainty while learning ecological cue validities. We therefore developed a diffusion lens model of vicarious functioning that captures dynamic cognitive adjustment to environments with multiple probabilistic and substitutable cues. A superstatistics approach allowed us to account for uncertainty reduction over time by an increasing sensitivity of the drift rate to the ecological validity of the cues. Additionally, the non-decision time is assumed to increase linearly with the number of presented cues to account for cue substitutability in the environment. The resulting model was validated by successfully fitting it to response time and choice data previously collected across multiple-cue probability learning tasks in diverse environments and scenarios. This suggests that the diffusion lens model can explain cognitive adjustment from an initial absence of knowledge to a near-perfect approximation of the probabilistic environment.

## Introduction

 

*Vicarious functioning is one of the most fundamental principles, if not the most fundamental principle, of behavior.* (Brunswik, 1957, p. 22)

Dynamic probabilistic environments impose two essential features on any knowledge, classification, or estimation task: uncertainty and substitutability (Brunswik, 1943; Gigerenzer & Kurz, 2001). The lack of a factual solution in such tasks inevitably introduces uncertainty about the correct answer. Thus, the individual must make do with the available error-prone proximal cues that are (more or less strongly) associated with the to-be-inferred distal criterion. This criterion can be represented by variable cue combinations, reflecting substitutability in the environment. Varying representations are perceived or retrieved from memory, but may be unavailable due to obscured perception or memory failure. The individual copes with these two characteristics with *vicarious functioning*, the principle of cognitive adjustment based on frequency of co-occurrences (Brunswik, 1952, 1955).

**Fig. 1:**
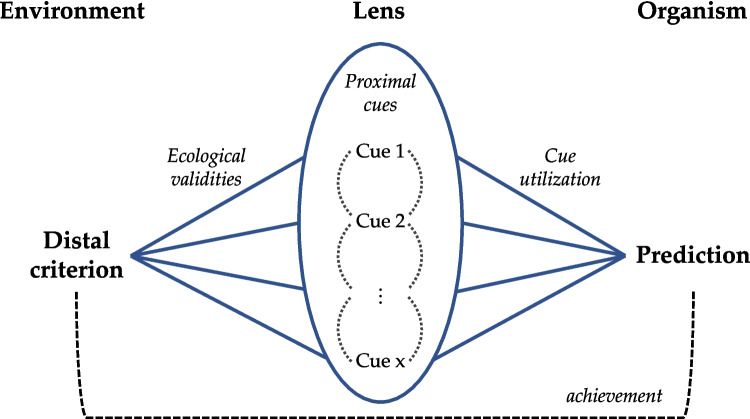
Illustration of the lens model (adapted from Brunswik, 1943; 1955)

For example, imagine a tourist being guided through Manhattan, New York City. This tourist is repeatedly asked to estimate the heights of skyscrapers. After giving each estimate, the tourist guide reveals the true height of the respective building. The tourist attempts to provide accurate estimates to the recurring question by leveraging multiple probabilistic cues, such as the counted number of floors in the building as well as the recalled height of the building the tourist lives in, and the estimated height of nearby skyscrapers, which can be used as a relative comparison. By adapting the estimate to the emerging cues in a changing environment, the tourist deals with uncertainty and substitutability in the height estimation task.

Vicarious functioning^1^ is essential to Egon Brunswik’s theory of probabilistic functionalism (Brunswik, 1934, 1943, 1955), which provides the framework for the present study. To our knowledge, there have been two approaches in previous research on how to model vicarious functioning. First, Brunswik (1955) himself proposed the measurement of vicarious functioning by correlational statistics as a means to directly quantify the association of probabilistic cues and the individual judgment. This analysis method was refined with the lens model equation (Hammond et al., 1964; Tucker, 1964). Second, Gerd Gigerenzer and colleagues developed the concept of *probabilistic mental models*, originally introduced to explain confidence in probabilistic inference tasks. This concept was later expanded to serve as the basis for the adaptive toolbox in judgment and decision-making (Gigerenzer et al., 1991, 1999; Gigerenzer & Kurz, 2001).

To foreshadow our critique of the lens model equation and probabilistic mental models, we argue that both approaches fail to account for dynamic cognitive adjustment over time, or represent no more than a special case of vicarious functioning with known ecological validities. Therefore, we advance a dynamic process model of vicarious functioning in probabilistic inference. Conceptually, we integrate Brunswik’s lens model with a dynamic diffusion decision model. Computationally, we validate the resulting diffusion lens model by fitting it to the choice and response time (RT) data from Scholten and Bröder (in press, Experiments 1–2). To this end, we apply a *neural superstatistics* approach (Schumacher et al., 2023, 2024) to estimate how the sensitivity to the ecological cue validities evolves with increasing experience of the environment.

### Probabilistic functionalism

Probabilistic functionalism is based on the assumption of an equal relationship between the environment and the individual (Brunswik, 1943, 1955). The theory aims to provide a general explanation of how individuals perceive and behave, that is, how they are “coming-to-terms” (Brunswik, 1957, p. 5) with their equal counterpart. Humans are conceived as intuitive statisticians who engage in inductive probabilistic inference (Brunswik, 1955; Doherty & Kurz, 1996). As illustrated in Fig. 1, the individual uses *proximal cues* (e.g., knowledge of the standard height of a skyscraper, relative heights of nearby buildings, etc.) as a “lens” to inductively infer a *distal criterion* in the environment that is not directly observable (e.g., the true height of a skyscraper). Two concepts describe how cues mediate between the distal criterion in the environment and the individual prediction: The *ecological validity* of a given cue is defined as the degree to which it accurately represents the distal criterion. By contrast, *cue utilization* concerns the weight that an individual assigns to a cue in the prediction of the distal criterion. The overall association of distal criterion value and prediction is called *achievement*, the objective of “proper cognitive adjustment” (Brunswik, 1957, p. 57). If, following repeated exposure, the hierarchy of cue utilizations aligns with the hierarchy of ecological validities, the individual achieves an accurate prediction of the distal criterion (Brunswik, 1955).

### Multiple-cue probability learning

To study probabilistic functionalism, *multiple-cue probability learning* (MCPL) has been developed (Brunswik & Herma, 1951; Hammond, 1955; Klayman, 1988). This experimental paradigm is consistent with Brunswik’s methodology of representative design (Brunswik, 1955).^2^ In MPCL, participants predict a criterion (e.g., a binary event such as win or loss) based on a given number of *k* cues, where each cue conveys a certain ecological validity across trials. Based on these validities, the participants receive feedback in each trial on whether their prediction was correct or incorrect. Specifically, the probability of an outcome given any combination of cues depends on the ecological validity of each cue and the intercorrelations among the cues.

Different versions of the MCPL paradigm have been developed (e.g., Adelman, 1981; Koele, 1980; Sniezek, 1986; Speekenbrink & Shanks, 2010), such as the weather prediction task, which allows the presence or absence of cues to vary (Gluck & Shohamy, 2002; Knowlton et al., 1994). In this task, 1 to $k-1$ cues are presented in each trial. For example, given a set of $k=4$ cues, one of 14 possible cue combinations appears in any single trial. As a result, this MCPL task emulates the real-world property of substitutability.

The basic concept of the lens model and tasks like cue probability learning entered various subfields of modern psychology, including research on judgment and decision-making (Brandstätter et al., 2006; Brehmer, 1994; Hammond et al., 1975; Karelaia & Hogarth, 2008), metacognition (e.g., Bröder & Undorf, 2019; Koriat, 1997), lie detection (e.g., Bond et al., 1994; Hartwig & Bond, 2011), cognitive neuroscience (e.g., Hopkins et al., 2011; Moustafa & Gluck, 2011), and psychological methodology (e.g., Dhami et al., 2004; Holleman et al., 2020). However, although previous lens model applications provided a comprehensive account of what individuals estimate, classify, or predict, the underlying mechanisms of how individuals arrive at these conclusions have essentially been overlooked. This was accompanied by a neglect of RTs in MCPL tasks (for an exception in a similar task, see Bröder & Gaissmaier, 2007), which we will argue to be sensitive markers of cognitive adjustment. Therefore, theory-guided modeling of RTs in MCPL tasks seems warranted to unravel cognitive adjustment processes in dynamic probabilistic environments.

### Uncertainty and substitutability

To understand the process of cognitive adjustment, it is important to consider the dynamic environment that the individual must adapt to Brunswik (1952). Specifically, the distal criterion is represented in the environment by substitutable proximal cues (*vicarious mediation*).^3^ That is, the environment produces variable combinations of proximal cues, which may be equally informative about the distal criterion. For example, when estimating the height of a skyscraper, the building may be partially occluded by another one from a certain vantage point; but instead, the typical height of a building in this district might come to mind again.

In general, the predictive accuracy of substitutable cues for the distal criterion is constrained by their ecological validity. Probabilistic error leads to uncertainty, caused by an incomplete state of knowledge (Juslin & Olsson, 1997; Juslin et al., 2007). However, uncertainty can be reduced when experiencing repeated occurrences of cue–outcome combinations. Uncertainty reduction is boosted by the interrelatedness of cues, which introduces redundancy into the environment (Dhami et al., 2004). Simply put, if the individual repeatedly receives the same feedback about an outcome when the cue is present, that outcome should be predicted the next time the cue is present.

Cognitive adjustment thus depends on the dynamics of cue substitutability in the representation of the criterion. Then, cognitive adjustment is the individual’s adaptive response to reduce uncertainty while coping with vicarious mediation in the environment. This process is known as *vicarious functioning*.

### Models of vicarious functioning

Previous modeling of vicarious functioning can essentially be divided into two approaches: the multiple-regression lens model (Hammond et al., 1964; Tucker, 1964) and the fast-and-frugal lens model (Gigerenzer & Kurz, 2001; Gigerenzer et al., 1991). In what follows, we contrast these two existing approaches with a new, more process-oriented approach: the diffusion lens model.

#### Multiple-regression lens model

Neo-Brunswikians claimed to capture vicarious functioning indirectly by modeling achievement. As a result, the primary objective of their research became the behavioral outcome (achievement) rather than the underlying process (cognitive adjustment; see Brehmer & Brehmer, 1988; Brehmer, 1994; Goldstein, 2004; Hammond et al., 1975; Klayman, 1988). Consistent with the assumption of an equal relationship between the environment and the individual, their conventional approach features separate linear models of the criterion and its prediction. Specifically, the environmental criterion $Y_e$ and the individual prediction $Y_i$ are conceived as linear functions of the same *k* cues $X_j$:

1 $$\begin{aligned} Y_e = \sum _{j=1}^{k} \beta _{e,j}X_j + \epsilon _e, \end{aligned}$$

2 $$\begin{aligned} Y_i = \sum _{j=1}^{k} \beta _{i,j}X_j + \epsilon _i, \end{aligned}$$

where $\beta _{e,j}$ denotes the association between the environmental criterion and a cue, $\beta _{i,j}$ the weight assigned by the individual to this cue, and $\epsilon _e$ and $\epsilon _i$ the respective error terms.

The lens model equation captures achievement by describing the alignment of these two linear models (for a comprehensive illustration, see Karelaia & Hogarth, 2008, p. 405). Specifically, the correlation $r_a$ between environmental criterion and individual judgment is defined as

3 $$\begin{aligned} r_a = G \, R_e \, R_i + C \sqrt{(1-R_{e}^{2})(1-R_{i}^{2})}. \end{aligned}$$

The matching index *G* quantifies the linear correlation between the predictions of the two models, $G = \rho _{\hat{Y}_e \hat{Y}_i}$. The multiple correlation coefficients $R_e$ and $R_i$ for the environmental and individual model reflect the environmental predictability ($R_e$) and the consistency of cue utilization ($R_i$). The parameter *C* denotes the correlation between the error terms of the environmental model and the individual model, that is, $C = \rho _{\epsilon _e\,\epsilon _i}$. If these errors are independent (i.e., $C=0$), the judgmental accuracy ($r_a$) is simply the product of matching (*G*), environmental predictability ($R_e$), and the individual’s response consistency ($R_i$) (Hammond et al., 1964; Karelaia & Hogarth, 2008; Sniezek, 1986). However, *C* may also deviate from 0 – for example, due to idiosyncratic associations (Bröder & Undorf, 2019) or configurality (Mellers, 1980).

A synthesis of 50 years of research on achievement in diverse decision-making contexts revealed that both environmental criteria and individual judgments are well described by these linear models (Brehmer, 1994; Karelaia & Hogarth, 2008; Klayman, 1988). In a meta-analysis of applications of the lens model equation encompassing 86 studies with 249 distinct environments, Karelaia and Hogarth (2008) concluded that participants in MCPL tasks are successful in reaching high achievement in non-noisy and even noisy environments, but inconsistent in applying their decision rule. This suggests that the experience of co-occurrences causes change in cue utilizations, reflecting an increasing alignment with the cues’ ecological validities over time.

Does any component of the lens model equation respect the temporal dynamics of cue utilization? While the consistency parameter $R_i$ and the non-linear parameter *C* may reflect some aspect of cognitive adjustment, they do not indicate the specific point in time when the individual predicts inconsistently or non-linearly and alters cue utilization. Therefore, we argue that the lens model equation and its extensions (Castellan, 1972; Stewart, 1976; Leipold et al., 2025) do not capture dynamic cognitive adjustment. Correspondingly, Karelaia and Hogarth (2008, p. 422) stated that “people use different combinations of cues across different trials (so-called vicarious functioning). Unfortunately, by estimating unique sets of weights for individuals across trials – and assuming that people are always applying the same weights – the linear lens model methodology does not capture this aspect of how people may be processing information.” Similar to Dhami et al. (2004, p. 970), the authors thus concluded that this multiple-regression lens model approach does not explain vicarious functioning.

#### Fast-and-frugal lens model

Gigerenzer et al. (1991) and Gigerenzer and Kurz (2001) identified two further reasons why multiple regression does not provide a satisfactory psychological model of the individual’s cognitive adjustment process: (1) its assumption of weighting and summing, and (2) the neglect of searching and stopping in decision-making. First, although multiple-regression models accurately fit experimental data, Gigerenzer and Kurz (2001) argued that the judgment process does not involve evaluating and carefully weighting all available cues. Why should people engage in this fine-balancing act if simpler strategies like unit weighting (Dawes, 1979, 1993) or fast and frugal heuristics (Gigerenzer et al., 1999; Gigerenzer & Selten, 2002) lead to a similar predictive accuracy? Second, multiple regression is silent about the order in which cues are searched and about when individuals stop their sampling process.

**Fig. 2:**
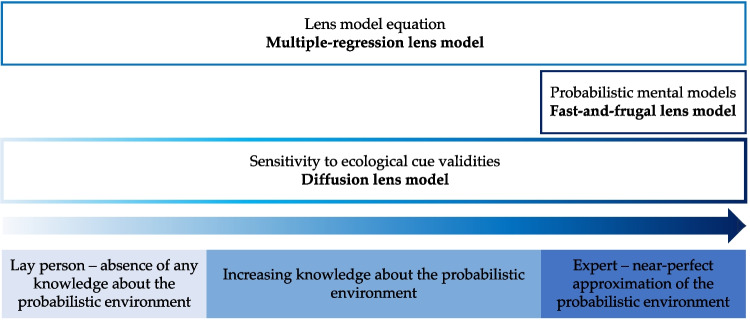
Illustration of a tripartite classification of knowledge over time in the context of repeated predictions. The *upper boxes* represent the level of expertise accounted for by the multiple-regression lens model, the fast-and-frugal lens model, and the diffusion lens model. The distinction between a transition and a non-transition in the colors of the boxes is indicative of the inclusion of cognitive adjustment in the model. The *arrow* signifies the increase in knowledge about the environment over time. The *lower boxes* depict three simplified states of knowledge based on experience with the probabilistic environment

Instead, Gigerenzer and colleagues put forth the idea of probabilistic mental models as the foundational theoretical construct for the so-called adaptive toolbox (Gigerenzer et al., 1991; Gigerenzer, 1993; Gigerenzer & Goldstein, 1996; Gigerenzer et al., 1999). If the factual solution to a binary question (e.g., which city has more inhabitants, *a* or *b*?) is unknown, a probabilistic mental model is used for inductive inference. That is, the target comparison activates a reference class (e.g., *a*, *b*, and similar cities) stored in long-term memory. Based on this reference class, which is a representative sample of further examples in a Brunswikian sense (Brunswik, 1955; Dhami et al., 2004), relevant probabilistic cues about the environment are generated from memory, to which corresponding validities can be assigned.

From a broader perspective, vicarious functioning should denote the flexible deployment of heuristics as adaptive tools (Gigerenzer & Kurz, 2001). In the exemplary case of a binary choice inducing the so-called take-the-best heuristic, cues may be generated and evaluated in the order of their ecological validity, with the most valid cue coming first. Given a probabilistic cue $C_j$ from the reference class *R*, answer *a* is chosen if the cue value assigned to answer *a* is larger than the cue value assigned to answer *b*. That is, in the terminology of Gigerenzer et al. (1991), answer *a* is chosen if

4 $$\begin{aligned} P(a\,|\,aC_jb;\,R) > P(b\,|\,aC_jb;\,R). \end{aligned}$$

However, if a cue does not distinguish between the two choice options, another cue is generated and subjected to the same evaluation procedure (Gigerenzer et al., 1991; Gigerenzer & Goldstein, 1996; Gigerenzer & Kurz, 2001). In a simplified situation in which cues are either present or absent, an option is selected if the cue with the highest ecological validity, which has not yet been evaluated, is present for that option but absent for the other option.

The fast-and-frugal lens model applies in decision situations in which an individual has already adjusted to a “known” environment based on familiar cues indicating a minimum of probabilistic error (Gigerenzer & Kurz, 2001). The authors “assume that cue validities correspond well to ecological validities” (Gigerenzer et al., 1991, p. 510). For example, in an MCPL task, the take-the-best heuristic may thus only apply after extensive practice, so that individuals have internalized ecological validities and can now utilize the hierarchy of ecological validities.

However, known environments constitute just one variant of a representative sample in a Brunswikian sense (Hammond, 1955; Lagnado et al., 2006; Speekenbrink & Shanks, 2010). The concept of representative samples is not exclusive to environments in which the individual has already undergone calibration and can act as an expert in that field (Fig. 2). Instead, the individual may also interact with the environment without any prior knowledge. Indeed, situations that require cognitive adjustment are common in real life, as everyone starts as a novice when acquiring new skills (e.g., learning to speak a language or to drive a car). Therefore, to investigate the reduction of uncertainty through experience, it is necessary that individuals lack knowledge of the environments’s structure at the beginning. If the environment is already known, the need to adjust to uncertainty is eliminated, and only substitutability remains to adjust to.

Empirical evidence lends support to the notion that the fast-and-frugal lens model only captures a special case of probabilistic decision-making (Bröder, 2000, 2003; Bröder & Schiffer, 2003; Juslin et al., 2003; Newell et al., 2003; Newell & Shanks, 2003). For example, Bröder (2000) demonstrated that only in strongly constrained environments with cue search costs, feedback, and successive cue displays more than $50\%$ of participants can be classified as users of the take-the-best heuristic. In all other environments tested, most participants applied a strategy of weighting and summing across cues. In addition, cue competition effects indicate that participants’ cue utilization – particularly in the absence of substantial knowledge about the environment – initially adheres to cue salience rather than ecological validity (Adelman, 1981; Kruschke & Johansen, 1999; Sniezek, 1986; Scholten & Bröder, in press).

### Diffusion lens model

Humans make decisions in probabilistic environments by relying on proximal cues that they consider to be associated with the distal criterion. These cues either stem from knowledge stored in memory or are immediately perceived as relevant to the current prediction. Cognitive adjustment then implies a change in cue utilization from one prediction scenario (or trial) to the next. This change is driven both by an internal adjustment based on the observation of co-occurrence of cues (i.e., based on experienced outcomes that lead to reduced uncertainty) and by an external adjustment brought about by the substitutability of the cues.

To account for vicarious mediation in the environment, the individual searches for currently available cues, guided by cue competition (e.g., Kruschke & Johansen, 1999) and salience (e.g., Scholten & Bröder, in press), and encodes them into a buffer. From the encoded pool of cues, evidence (i.e., information that supports an answer) is sampled until the accumulated evidence reaches the decision threshold for a prediction about the criterion. For example, accumulated evidence might stem from surface features or ecological validities read from the cues. If a decision threshold is reached, the corresponding motor response is prepared and executed. Cue utilization reflects the evidential strength of the sampled information: the more strongly a sampled cue supports one decision, the more rapidly the evidence accumulates towards the corresponding decision threshold.

Importantly, this speed of information uptake is thought to be determined by the individual’s sensitivity to the ecological cue validity. In the one extreme of complete uncertainty, the sensitivity is zero, and decision-making amounts to a guessing process, which may be decided by sampling from invalid cues (biased drift) or by chance alone (zero drift). In the other extreme of an established alignment with the ecological cue validities, the speed and accuracy of the decision process is driven by how extreme the overall ecological validity of the available cues is. The diffusion lens model thus accounts for uncertainty reduction over time by allowing sensitivity to increase with experienced co-occurrences.

Additionally, the model also allows the separation between the two decision thresholds (assuming a binary choice) and the non-decision time to systematically change across trials, considering that both parameters have often been found to decrease with practice in other tasks (e.g., Dutilh et al., 2009; Evans & Brown, 2017; Kelber et al., 2025). Furthermore, in probabilistic environments, the “decision caution” (i.e., threshold separation) may not only reflect one’s accuracy motivation but also the experienced noise in the task. This also suggests that individuals become less cautious as they grow more familiar with the environmental structure behind the noisy feedback.

Which qualitative predictions can be drawn from the assumptions of the diffusion lens model? First, the more cues are presented per trial, the longer the stimulus encoding prior to decision-making and thus the longer the non-decision time and the RT. Second, the drift rate increases with ecological cue validity, which should in turn shorten RT and boost accuracy. Third, greater experience with the environment enhances the sensitivity to the ecological cue validities and thus the drift rate, leading to shorter RT and higher accuracy. Fourth, even with marginal experience with the environment, sensitivity should be higher when salient features of the cues match their ecological validity, leading to a higher drift rate, shorter RT, and higher accuracy. For example, ecological validities should be more salient when positively (negatively) valenced cues are associated with a positive (negative) outcome rather than vice versa (Scholten & Bröder, in press). These considerations suggest that RTs in MCPL tasks are sensitive markers of cognitive adjustment processes. However, note that RTs may change for a variety of reasons – for instance, shorter RTs could be due to higher drift rate, lower threshold separation, shorter non-decision time, or some combination. We therefore proceed to a formalization of the diffusion lens model, which will later allow us to separate changes in the different process components by fitting the computational model simultaneously to choice and RT data from MCPL tasks.

## Model specification

The diffusion lens model links the temporal dynamics of decision-making under uncertainty that are assumed to operate on two distinct time scales. The judgment process within each trial is represented by a diffusion decision model (DDM; Ratcliff, 1978; see also Ratcliff et al., 2016; Voss et al., 2013), modified to account for probabilistic inference in multiple-cue environments. Moreover, to capture cognitive adjustment over time, the exploratory superstatistics approach (Schumacher et al., 2023, 2024) allows selected model parameters to systematically change from trial to trial. Superstatistics provides a general framework for modeling systems whose dynamics unfold on multiple time scales. It assumes that observable data arise from a (low-level) stochastic process whose parameters themselves evolve more slowly according to another (high-level) stochastic process. This hierarchical formulation captures non-stationarity and heterogeneity in complex systems, offering a principled way to model gradual contextual or cognitive adjustments over time, such as uncertainty reduction during probabilistic learning in the present study.

The diffusion lens model thus establishes a two-level hierarchy of stochastic processes, which can be formalized as:

5 $$\begin{aligned}&\mathbf {Low\!\!-\!\!level~model~component}\nonumber \\ x_t&\sim \text {wiener}(v_t, a_t, \tau _t, \beta ) \end{aligned}$$

6 $$\begin{aligned} v_t&= v_0 + b_{v, t} * \text {cue validity} \end{aligned}$$

7 $$\begin{aligned} \tau _t&= \tau _{0, t} + b_{\tau } * n_{\text {cues}} \end{aligned}$$

8 $$\begin{aligned}&\mathbf {High\!\!-\!\!level~model~component}\nonumber \\ b_{v, t}&= b_{v, t-1} + \sigma _v \xi _t \end{aligned}$$

9 $$\begin{aligned} a_t&= a_{t-1} + \sigma _a \xi _t \end{aligned}$$

10 $$\begin{aligned} \tau _{0, t}&= \tau _{0, t-1} + \sigma _{\tau } \xi _t \end{aligned}$$

11 $$\begin{aligned} \xi _t&\sim \mathcal {N}(0, 1) \end{aligned}$$

Within each trial *t*, the data *x* (binary choice and RT) are generated by a Wiener process. This noisy evidence-accumulation process drifts at an average rate of $v_t$ (drift rate) between two absorbing boundaries, which represent the possible decisions (e.g., truth or lie). When the accumulated evidence reaches the upper threshold $a_t$ or the lower threshold 0, the judge decides in favor of the alternative supported by the evidence. Evidence accumulation begins from the relative starting point $\beta$, which may but need not be 0.5, to account for a priori decision biases towards one of the two outcomes (e.g., positivity bias towards truth, or negativity bias towards lie). Finally, the non-decision time $\tau _t$ is added to the decision time to account for the duration of decision-unrelated processes, such as stimulus encoding and response execution. Taken together, the data-generating Wiener process builds on four parameters (see Eq. 5).

**Table 1:** Ecological cue validities in the environments created by Scholten and Bröder (in press, Experiments 1–2)

Cue							Outcome		
Pattern	1	2	3	4	Total	*P*(Pattern)	*a*	*b*	*P*($a\,|\,$Pattern)
A	1	1	1	0	23	0.093	21	2	0.913
B	1	1	0	1	11	0.045	9	2	0.773
C	1	1	0	0	32	0.130	29	3	0.906
D	1	0	1	1	11	0.045	3	8	0.227
E	1	0	1	0	15	0.061	12	3	0.800
F	1	0	0	1	8	0.033	4	4	0.500
G	1	0	0	0	23	0.093	21	2	0.913
H	0	1	1	1	23	0.093	2	21	0.087
I	0	1	1	0	8	0.033	4	4	0.500
J	0	1	0	1	15	0.061	3	12	0.200
K	0	1	0	0	11	0.045	6	5	0.546
L	0	0	1	1	32	0.130	2	30	0.063
M	0	0	1	0	11	0.045	5	6	0.454
N	0	0	0	1	23	0.093	2	21	0.087
Total					246	1.000	123	123	

*Note.* Face absent $= 0$, face present $= 1$. The cue patterns 1111 (all faces present) and 0000 (no face present) were not used. The “Total” column indicates the total number of times a pattern appears in one session of 246 trials. The “Total” row shows, in order, the sum of the trials, the total probability of occurrence of the patterns, and the sum of the absolute numbers for the positive outcomes (truth/win; *a*) and negative outcomes (lie/loss; *b*). “P(Pattern)” indicates the unconditional probability of occurrence for a pattern, and “P($a\,|\,$Pattern)” the probability of a positive outcome given a specific pattern. The frequency distribution was taken from Lagnado et al. (2006) and adapted to a design with 246 trials

The drift rate in each trial, $v_t$, is modeled as a linear function of the overall ecological validity of the cues presented in this trial (Eq. 6), where $b_{v, t}$ measures the sensitivity to the ecological cue validity and $v_0$ represents a base drift rate. This base drift rate accounts for dynamic biases arising when an individual mistakenly also samples evidence from invalid cues that are unrelated to the ecologically valid cues.^4^ Analogously, the non-decision time $\tau _t$ is modeled as a linear function of the number of presented cues $n_{\text {cues}}$ (Eq. 7), where $\tau _{0, t}$ reflects a base non-decision time required for cue-independent processes (e.g., motor execution) and $b_\tau$ measures the additional non-decision time needed for encoding each presented cue. Due to these modifications compared to the DDM, the low-level component of the diffusion lens model accounts for the two cornerstones of Brunswikian probabilistic functionalism. First, the non-decision time accounts for vicarious mediation (i.e., substitutable cues in the environment) by changing with the number of to-be-registered cues in each trial. Second, the drift rate accounts for uncertainty (inversely related to achievement).

Across trials, three lower-level model parameters are allowed to vary according to a Gaussian random walk (Eq. 8, 9, 10, and 11). This introduces three high-level parameters $\eta = \{\sigma _v, \sigma _a, \sigma _{\tau }$} that quantify the extent of overall parameter fluctuation (i.e., the standard deviation, SD, of the Gaussian random walk noise). Specifically, we allowed for changes in the sensitivity of the drift rate to the ecological cue validity ($b_{v, t}$) to account for uncertainty reduction over time. In addition, decision threshold ($a_t$) and base non-decision time ($\tau _{0, t}$) could also vary to account for practice-induced decreases in decision caution and decision-unrelated speed-ups. For parsimony, we did not further allow for across-trials variations in base drift rate ($v_0$), relative starting point ($\beta$), or additional non-decision time per cue ($b_\tau$).

In summary, both levels of the diffusion lens model provide different but complementary theoretical contributions. At the low level, the Brunswikian principles of uncertainty and substitutability are built into a DDM: The drift rate reflects the sensitivity to the ecological validities and thus combines uncertainty in the environment and in the individual. The non-decision time is adapted to the number of presented cues to account for the implication of environmental substitutability that different numbers of cues are available on different occasions. At the high level, cognitive adjustment processes are captured by allowing some parameters to vary flexibly across trials with a superstatistics approach. The across-trials dynamics of the present diffusion lens model are therefore not constrained by a specific learning mechanism or updating rule. Instead, a central goal of our modeling approach is to explore whether the model successfully recovers uncertainty reduction – a foundational principle of adaptive probabilistic learning – via an increasing sensitivity to the ecological cue validities.

## Model validation

### Study details

**Fig. 3:**
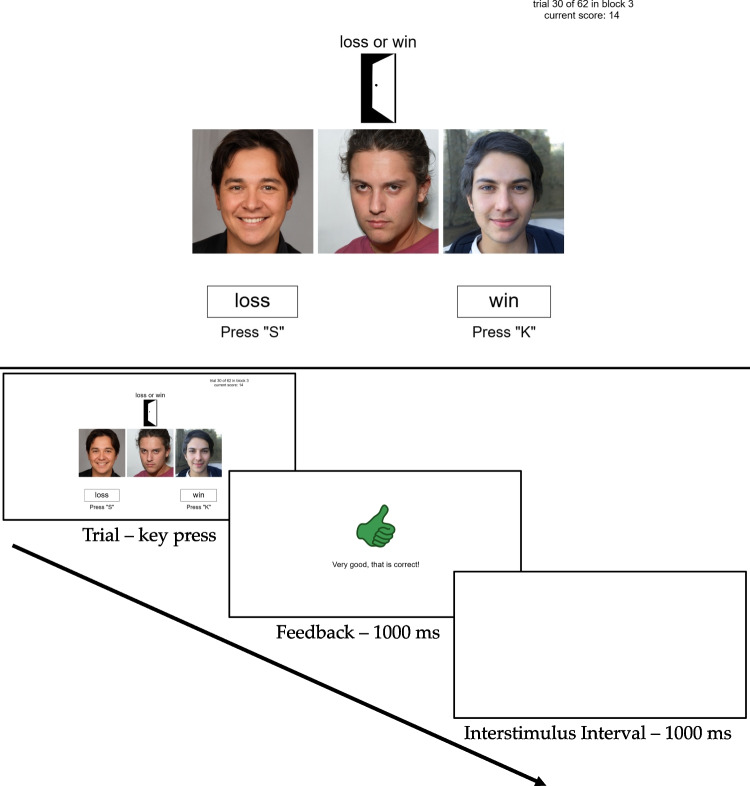
Example screen (*upper panel*) and trial course (*lower panel*) for the MCPL tasks in Scholten and Bröder (in press, Experiments 1–2). The faces were adapted from the One Million Impressions database (Peterson et al., 2022)

To evaluate the diffusion lens model, we employed the choice and RT data from the two experiments ($N_{\text {Experiment 1}} = 98$ participants, $N_{\text {Experiment 2}} = 160$ participants) conducted by Scholten and Bröder (in press). Each participant completed 246 trials of a modified weather prediction task (Gluck & Shohamy, 2002; Lagnado et al., 2006). In this MCPL task, four faces served as cues in 14 different cue combinations. Table 1 shows the conditional probabilities for the binary outcome given each of these 14 patterns. In both experiments, the four faces were associated with the positive outcome with the probabilities 0.2, 0.4, 0.6, and 0.8. Thus, two cues were strongly predictive and two cues were weakly predictive, with one cue tending to the positive outcome and one to the negative outcome. Each trial featured one, two, or three faces (for an example screen and trial course, see Fig. 3). These faces were adapted from the One Million Impressions face database (Peterson et al., 2022). The binary outcome was dichotomized as positive or negative in two scenarios: “truth” versus “lie” in a witness scenario (Experiments 1–2), and “win” versus “loss” in a gambling scenario (Experiment 2).

**Table 2:** Mean correct RT as a function of number of cues and congruence in Experiments 1–2 from Scholten and Bröder (in press)

Experiment (scenario)	Congruence	1 cue	2 cues	3 cues
Experiment 1 (witness)	Congruent	1.218 (0.247)	1.566 (0.379)	1.926 (0.532)
	Incongruent	1.622 (0.484)	2.008 (0.551)	2.413 (0.751)
Experiment 2 (witness)	Congruent	1.083 (0.310)	1.335 (0.449)	1.602 (0.521)
	Incongruent	1.300 (0.428)	1.633 (0.564)	1.969 (0.663)
Experiment 2 (gambling)	Congruent	1.057 (0.438)	1.319 (0.529)	1.614 (0.733)
	Incongruent	1.219 (0.266)	1.511 (0.356)	1.803 (0.454)

*Note.* The SD is provided in brackets. All RT values were rounded to the nearest millisecond

The salience of the ecological cue validities was manipulated between participants by matching versus mismatching the validities to the valence of the faces. Specifically, in the congruent environment, the presentation of more (less) trustworthy faces was associated with the positive (negative) outcome. This mapping was reversed in the incongruent environment. For subsidiary details on the experiments, see the associated online repository (https://osf.io/23gkz). All code and data of this study are available in our GitHub repository at https://github.com/LuSchumacher/vicarious-functioning-nsddm.

Scholten and Bröder (in press) examined the impact of facial trustworthiness as a valence-defining, task-irrelevant cue on decision-making strategies over time. To illustrate the learning trajectories in congruent and incongruent environments, the authors compared the utilization of cues based on their salience and the individual’s learning experience. The performance analyses from Scholten and Bröder (in press) used multiple regression and can be classified within the traditional lens model approach initially discussed. In contrast, the present study not only employed choices but also previously unanalyzed RTs to uncover the underlying cognitive adjustment process by validating a new model of vicarious functioning.

**Fig. 4:**
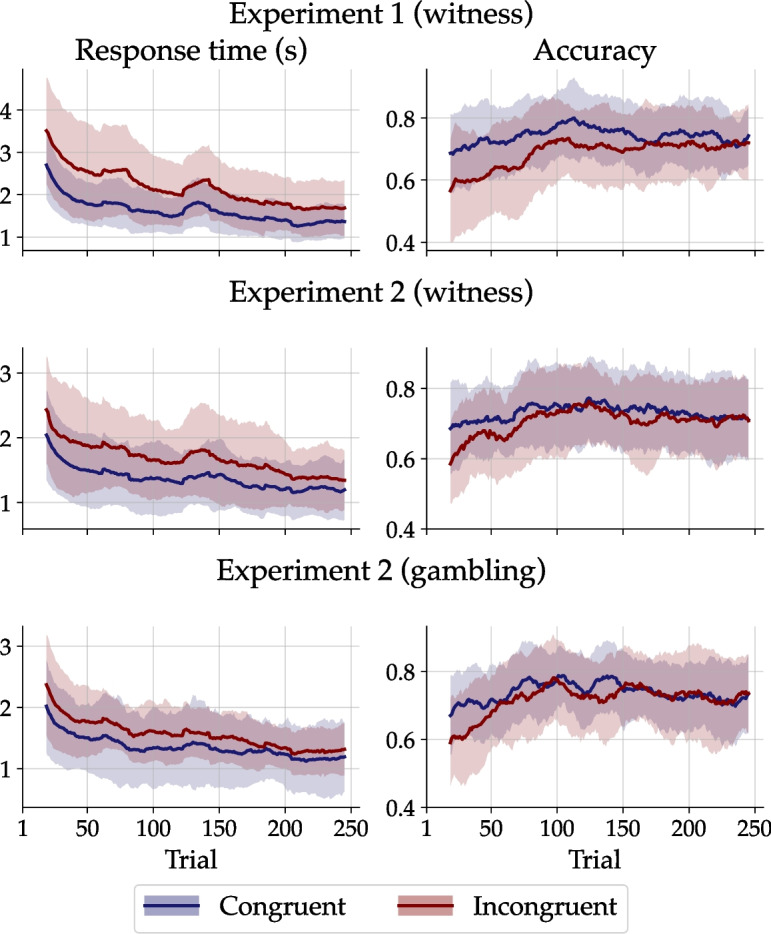
Observed RT and accuracy series. The *blue* and *red lines* represent the data smoothed using a simple moving average with a lag of 20 trials, averaged across individuals for the congruent and incongruent conditions, respectively. *Shaded areas* indicate the SD across participants

Several experimental details lend themselves to the validation of the diffusion lens model. Most importantly, participants started the task without any knowledge about the ecological cue validities and subsequently underwent 246 trials, during which achievement stabilized according to the behavioral analyses by Scholten and Bröder (in press). This enabled us to model how cognitive adjustment evolves from an initial state of complete uncertainty to a near-perfect approximation of the probabilistic environment (see Fig. 2). Moreover, the manipulation of salience permits an investigation of vicarious functioning in environments that are supportive versus adverse to the learning of the cue validities. Finally, the witness scenario in Experiment 2, which otherwise replicates Experiment 1 with Prolific workers instead of students, allowed us to check the robustness of the model validation results.

### Behavioral data

**Fig. 5:**
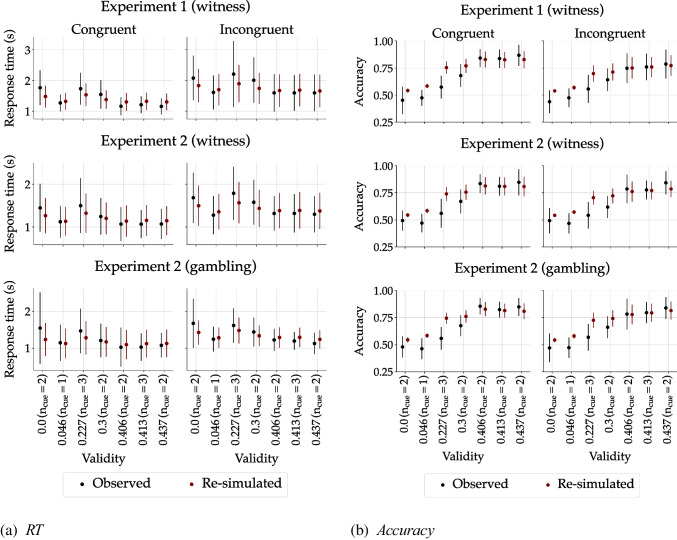
Observed and re-simulated **a** RTs and **b** accuracies. *Points* represent the means across participants, with *error bars* indicating the SD. Observed data are shown in *black* and re-simulated data in *red*. The *x*-axes represent the ecological validity as $|P(a\,|\,\text {Pattern}) - 0.5|$, so that larger values reflect higher ecological validity

Henceforth, we summarize the group-level RT and accuracy data that pertain to the qualitative predictions drawn from the core assumptions of the diffusion lens model above. First, RT increased with the number of presented cues (see Table 2). Second, with increasing ecological cue validity, performance generally improved, with RT tending to decrease and accuracy tending to increase (Fig. 5a). Note that the patterns on the x-axes in Fig. 5a differ not only in ecological validity but also in the number of cues, which influence RT as well (see above). Third, the larger the practice, the better the performance, as reflected in decreasing RT and increasing accuracy (see Fig. 4). Fourth, RT was lower and accuracy higher when the environment was congruent rather than incongruent (see Table 2 and Fig. 4). These general patterns held true across scenarios (witness and gambling) and environments (congruent and incongruent). Taken together, the behavioral data corroborate all qualitative predictions of the diffusion lens model.

### Model fit

To fit our model to each individual data set from all three experiments, we employed Amortized Bayesian Inference (ABI; Bürkner et al., 2023; Radev et al., 2020), a modern inference technique that leverages neural networks to efficiently approximate posterior distributions. ABI comprises two stages: a training phase, in which the networks learn a surrogate posterior from simulations of the generative model, and an inference phase, in which they estimate model parameters for new data sets consistent with the model. Notably, this approach circumvents the need for an explicit likelihood function or numerical integration. Following the procedure outlined by Schumacher et al. (2023), who demonstrated that ABI outperforms traditional Bayesian methods for complex cognitive models, we trained our networks using only simulations from the generative model. For details on the specific implementation used here, see Appendix A.

After training, we obtained 2000 posterior samples for each individual. To evaluate model fit, we conducted posterior re-simulations. Specifically, we used 500 draws from each individual’s posterior distribution and simulated choice and RT data from the generative model. These re-simulations were then aggregated by computing the median predictions (along with mean and SD) across participants, separately for each cue validity level and experimental condition. Figure 5 displays the resulting posterior re-simulations alongside the observed group-level data.

It can be seen that the diffusion lens model provides a good fit to the observed data across all environments and scenarios. The model tracks the general data patterns, especially in RTs but also in accuracy, demonstrating its capacity to capture both the general trends and also finer details of decision-making behavior for varying levels of cue validity. Although the general alignment between observed and re-simulated data supports the validity of the diffusion lens model, some slight misfits are also evident. In particular, the model tends to overestimate the judgment accuracy at one level of ecological cue validity (i.e., 0.227).

**Fig. 6:**
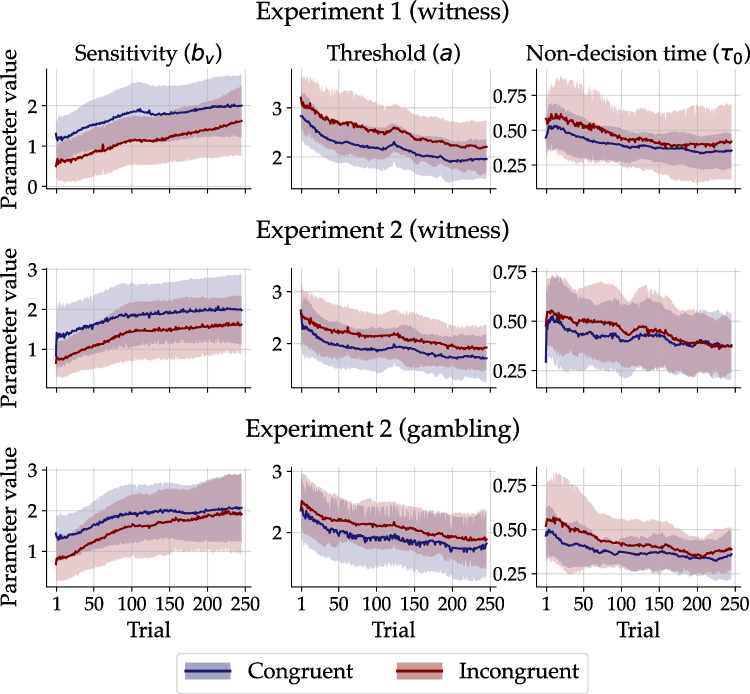
Estimated parameter trajectories. The *solid blue* and *red lines* represent the median posterior estimates of the three non-stationary model parameters ($b_v$, *a*, and $\tau _0$), averaged across individuals at each time step for the congruent and incongruent conditions, respectively. The *shaded regions* indicate the SD of the median posterior distributions

### Parameter estimates

Figure 6 depicts the inferred parameter trajectories, which were computed by taking the median of each participant’s posterior distribution and then aggregating the results across participants (mean and SD) separately for each experimental condition. Crucially, the sensitivity of the drift rate to the ecological cue validity ($b_v$) increased over time in all environments and scenarios. The diffusion lens model thus successfully recovered the reduction of uncertainty with increased experience of cue-outcome co-occurrences, as postulated in Brunswik’s theory of probabilistic functionalism. Furthermore, the sensitivity to the cue validity was generally higher in congruent as opposed to incongruent environments. Only for the gambling scenario, participants achieved similar levels of sensitivity in incongruent and congruent environments towards the end of the session.

As expected, both threshold separation (*a*) and base non-decision time ($\tau _0$) decreased over time in all environments and scenarios. This suggests that increased experience made participants less cautious in their decision-making and faster in cue-unrelated non-decision processes. Interestingly, participants tested in incongruent (versus congruent) environments tended to be more cautious in decision-making and slightly slower in cue-unrelated non-decision processes (e.g., motor execution). Note that the estimated parameter trajectories for the first few trials sometimes did not follow these otherwise robust trends (see, e.g., witness condition in Experiment 2). This is likely due to measurement error for the initial trials and thus should not be given further interpretation (Alister & Evans, 2025).

**Fig. 7:**
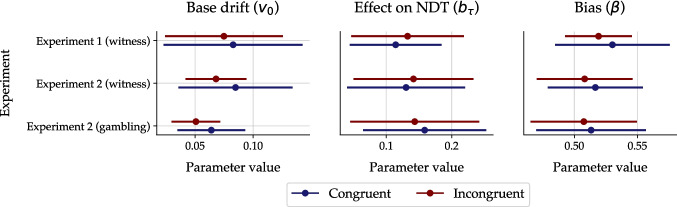
Estimated static parameters. Each *point* represents the median of the posterior distribution, averaged across participants, with *error bars* indicating the SD. NDT = non-decision time

In addition to the three non-stationary parameters, we also estimated three parameters that were not allowed to vary over time. Figure 7 presents the posterior medians of these static parameters, aggregated across individuals (mean and SD) and separately for each experimental condition. For the base drift rate ($v_0$), which represents a cue-unrelated dynamic bias in mental speed, the posterior distributions were concentrated near zero in all environments and scenarios. This confirms that the sensitivity to the ecological cue validity is the primary driver of decision-making in probabilistic environments. Moreover, the estimates of $b_\tau$ indicate that decision-unrelated processes take about 150 ms longer per additional cue, which likely reflects the time needed to encode one face. Finally, the average posterior medians for the relative starting point ($\beta$) were only slightly above 0.5. This suggests that there was, at most, a small a priori bias towards the positively valenced outcome (truth/win). Interestingly, both $\beta$ and $v_0$ tended to be slightly higher in participants tested in congruent (versus incongruent) environments, which might hint at small a priori and dynamic positivity biases evoked by supportive (versus adverse) environments.

## Discussion

The theory of probabilistic functionalism (Brunswik, 1952, 1955) posits a dynamic inference process, in which the individual adapts the utilization of proximal cues during a series of changing environmental representations to achieve an accurate prediction of the distal criterion. To capture this cognitive adjustment, termed vicarious functioning (Brunswik, 1957), we developed a diffusion lens model that allows the rate of evidence accumulation in a binary decision process to increasingly align with the ecological cue validities over time. In addition, the model regards the non-decision time as a linear function of the number of presented cues. Accordingly, the processes underlying individual achievement are shaped by the ecological validity of the presented cues, shifting cue patterns, and the experience with the environment.

This novel cognitive process model was validated against choice and RT data previously collected in a modified weather prediction task (Scholten & Bröder, in press, Experiments 1–2). In this MCPL task, participants had to adapt to the ecological validities of four intermittently presented faces to improve the accuracy of their binary judgments in a witness scenario (truth or lie) or a gambling scenario (win or loss). More (less) trustworthy faces were associated with truth/win (lie/loss), or vice versa, thus rendering the environment congruent or incongruent. Despite its simplicity, the diffusion lens model fitted these data well, suggesting that it adequately accounts for MCPL across different scenarios and environments.

What are the boundary conditions of the postulated process of adapting to the overall ecological validity of the cues based on the frequency of co-occurrences? As the posterior re-simulations have revealed, the diffusion lens model accounted well for judgment accuracy and RTs across most cue combinations from low to high ecological validity. However, the model overestimated the accuracy for the two patterns B and D with an ecological validity of about 0.227, which involve two faces strongly and weakly indicating one outcome and one face strongly indicating the other (see Table 1 and Fig. 5). This misfit suggests that when two cues are strongly associated with opposing outcomes, individuals struggle to identify a relatively high overall ecological validity caused by a third cue that is weakly associated with one of the two outcomes. Therefore, the current formalization of the diffusion lens model may not accurately capture the decision process when a weak cue drives the overall ecological validity because stronger cues point in opposite directions and thus balance each other out.

The evidence-accumulation process proposed to underlie performance within each trial was supplemented by an exploratory superstatistics approach (Schumacher et al., 2023, 2024) that allowed selected model parameters (sensitivity to cue validity, decision caution, base non-decision time) to change from trial to trial. Most importantly, the sensitivity of the drift rate to the ecological cue validity increased over time to a certain asymptote. The trajectories also revealed that decision caution and non-decision time decreased asymptotically over time, which is consistent with previous findings in many other tasks (e.g., Dutilh et al., 2009; Evans & Brown, 2017; Kelber et al., 2025). Taken together, these wide-ranging cognitive adjustments indicate the need for dynamic modeling of choices and latencies to capture uncertainty and substitutability in Brunswikian environments (see also Busemeyer et al., 1993; Diederich, 1997; Juslin & Olsson, 1997).

Previous models examining decision making in probabilistic environments include the multiple-regression lens (Brehmer, 1994; Hammond et al., 1964; Karelaia & Hogarth, 2008) and the fast-and-frugal lens (Gigerenzer & Goldstein, 1996; Gigerenzer et al., 1991). The multiple-regression approach in the form of the lens model equation assumes that probabilistic predictions are a function of summed cue weightings (for a hierarchical extension of this approach, see Leipold et al., 2025). Thus, many Neo-Brunswikians fitted multiple-regression models to accuracy data from cue probability learning tasks to represent overall cue utilization and obtain one behavioral measure of achievement (Hammond et al., 1964; Karelaia & Hogarth, 2008). Although this approach may be convenient and sufficient for many practical applications, the lens model equation overlooks vicarious mediation as well as the temporal dynamics of sequential judgments. In contrast, the probabilistic mental models approach (Gigerenzer et al., 1999) proposes that individuals make use of a strict hierarchy of cues’ ecological validities. Then, decisions follow simpler heuristics adaptive to changing cue availability in the environment. However, these theoretical assumptions not only seriously constrain the distinctiveness of situations, but also the empirical prevalence of people applying the fast-and-frugal lens (e.g., under strong environmental constraints; Bröder, 2000).

By accounting for within-trial and across-trial dynamics, the diffusion lens model departs from the multiple-regression and fast-and-frugal lens models. Going beyond previous approaches to capture vicarious functioning, the low-level model component (within-trial diffusion process) incorporates uncertainty by linking the drift rate to the sensitivity to the ecological cue validities, and also accounts for variable cue combinations, implied by substitutability, by linearly increasing the non-decision time with the number of presented cues per trial. In addition, the high-level model component (across-trials superstatistics approach) can account for uncertainty reduction – a foundational principle of adaptive probabilistic learning. Consequently, both model components offer distinct theoretical advancements within an integrated two-level architecture.

In an empirical comparison of Gigerenzer’s adaptive toolbox approach and diffusion decision models (DDMs), Newell (2005); Newell and Bröder (2008) have argued convincingly that DDMs provide a more comprehensive representation of decision processes. These models can capture the full spectrum of individual heuristics caused by different states of knowledge within a single, simple mechanism, offering a more holistic and integrated perspective on human decision-making. In particular, our diffusion lens model delineates cognitive adjustment from complete uncertainty (due to the absence of relevant knowledge) to alignment with the ecological cue validities. This across-trial dynamics extends the within-trial dynamics in the model, which conceptualizes the judgment process as sequential sampling of evidence from the pool of available cues.

The diffusion lens model is, to our knowledge, the first process model that captures cognitive adjustment over time as postulated by Brunswik’s theory of probabilistic functionalism. However, the model is comparable to previous reinforcement-learning DDMs (RL-DDMs) for multiple-armed bandit tasks. In these models, the subjective values for the available choice options are updated according to some learning rule and mapped onto the drift rate of the decision process. Pedersen et al. (2017) and Fontanesi et al. (2019) applied RL-DDMs to value-based decision-making tasks, in which participants received probabilistic rewards when choosing one of two presented stimuli. But note that the representation of the environment in MCPL and RL tasks differs substantially. Whereas MCPL requires the individual to predict a distal criterion in the environment by utilizing proximal cues as mediators, RL involves the comparison of distinct choice options with implicit reward values. Consequently, the interpretation of the learning rate for RL-DDMs is constrained to the extent that outcome frequency judgments correspond to the memorized reward values that accompany the different options. Conversely, uncertainty reduction in the modified weather prediction task is predicated on the intercorrelation of shown cue patterns with a single distal entity, yielding two possible outcomes.

Similarly, Sewell et al. (2019) and Sewell and Stallman (2020) modeled data from a single-cue probability learning task, which required a binary category classification based on one cue per trial and the probabilistic category feedback from the previous trials. The resulting choice and RT data were well fitted by DDMs, whose drift rate for the more consistent cues (probabilities of 0.2 and 0.8 for one category) and the less consistent cues (0.4 and 0.6) followed an exponential distribution (descriptive approach) or an error-based updating rule (mechanistic approach). While single-cue probability learning incorporates cues mediating to a distal criterion, these studies did not examine the learning process based on the interrelatedness of all existing cues. Instead, a similar approach to RL-DDMs is evident, wherein the cues are regarded as discrete entities, each exhibiting a propensity to gravitate towards a specific outcome. MCPL entails a more complex learning environment, as the integration of multiple cues can yield a pattern that signifies either conflicting outcomes or a consolidation of one outcome. Participants achieve not only the ecological validity of distinct single cues, but also the intercorrelations between cues, which again requires dynamic cognitive adjustment. Consequently, the present study generalizes the findings of Sewell et al. (2019) and Sewell and Stallman (2020) to a multiple-cue environment. In a stable and symmetrical environment with two cues mapping strongly or weakly onto one outcome, respectively, the diffusion lens model captures learning by pattern-outcome co-occurrences. To further refine the understanding of how conflicting or converging cue patterns moderate vicarious functioning, future studies may examine uncertainty reduction in an environment with more cues that exhibit extreme ecological validity.

The present study used the exploratory superstatistics framework to investigate the across-trial dynamics in multiple-cue environments. This approach has been validated in its ability to recover the cognitive process dynamics, even when interindividual variance is high, such as for the diverse samples and the modified weather prediction task used. Future studies could make use of this feature of the superstatistics approach to model cognitive adjustment in response to gradual versus sudden shifts of ecological cue validities in dynamic environments, as implemented for the multiple-regression lens model by Speekenbrink and Shanks (2010). Moreover, follow-up research may also complement the present exploratory approach by constraining the cognitive across-trials dynamics to follow a learning mechanism (e.g., Fontanesi et al., 2019; Pedersen et al., 2017; Sewell et al., 2019) or a plausible distribution (Alister & Evans, 2025; Sewell et al., 2019).

For parsimony, uncertainty reduction in multiple-cue environments was captured by the trajectory of a single parameter, which reflects the sensitivity of the drift rate to the overall ecological validity of the presented cues. Indeed, the proposed basic form of the diffusion lens model was validated by corroborating its plausible qualitative predictions and demonstrating its satisfactory quantitative fits to RT and accuracy data from MCPL tasks. We hope that our initial model formulation stimulates the development and comparison of further variants of the diffusion lens model that explain uncertainty reduction via specific learning mechanisms and thereby make even narrower predictions. Refined process models of vicarious functioning may also specify how each individual cue contributes to the drift rate. Ideally, these models could implement different strategies (e.g., multiple-cue, single-cue, or singleton; see Gluck et al., 2002) or different assumptions about when individuals shift between these strategies. Comparisons of such models could further accelerate theoretical progress, analogous to previous comparisons of multiple-regression and fast-and-frugal lens models (Dhami & Harries, 2001). Future theory-guided modeling may reveal whether the diffusion lens model – or some variation thereof – can fulfill the role of the longed-for Brunswikian model of judgment under uncertainty (Dhami et al., 2004; Juslin et al., 2007), in the sense of Brunswik’s naïve statistician.

## Data Availability

Materials are available at https://osf.io/23gkz and data are available at https://github.com/LuSchumacher/vicarious-functioning-nsddm.

## Appendix A Amortized Bayesian inference

The goal of our deep learning approach is to infer the joint posterior distribution over three time-varying low-level model parameters $\theta _{1:T} = \{b_{v,1:T}, a_{1:T}, \tau _{0,1:T}\}$, three time-invariant low-level parameters $\phi = \{v_0, \tau _0, \beta \}$, and three static high-level parameters $\eta = \{\sigma _v, \sigma _a, \sigma _{\tau }\}$. Thus, we aim to recover the full joint posterior $p(\theta _{1:T}, \phi , \eta \mid x_{1:T})$ from the observed time series of choices and RTs ${x_t}_{t=1}^T$:

12 $$\begin{aligned} p(\theta _{1:T}, \phi , \eta \mid x_{1:T}) \propto p(\theta _0, \phi , \eta ) \prod _{t=1}^T p(x_t \mid \theta _t, \phi ) \prod _{t=1}^T \mathbb {T}(\theta _t \mid \eta , \theta _{t-1}), \end{aligned}$$

where $p(\theta _0, \phi , \eta )$ is the joint prior over the initial time-varying parameters, time-invariant parameters, and high-level hyperparameters. The transition model $\mathbb {T}$ encodes the temporal dynamics of the low-level parameters via a Gaussian random walk:

13 $$\begin{aligned} \mathbb {T}(\theta _{k,t} \mid \theta _{k,t-1}, \sigma _k) = \mathcal {N}(\theta _{k,t} \mid \theta _{k,t-1}, \sigma _k), \end{aligned}$$

where *k* indexes the individual model parameter. We placed the following priors on the initial values of the time-varying parameters, the time-invariant parameters, and the high-level model parameters:

$$\begin{aligned} b_{v, 0}&\sim \mathcal{T}\mathcal{N}(0, 2.5, 0.0, 6.0), \\ a_{0}&\sim \mathcal{T}\mathcal{N}(2.5, 1.0, 0.0, 6.0), \\ \tau _{0, 0}&\sim \mathcal{T}\mathcal{N}(0.3, 0.25, 0.0, 2.0), \\ v_0&\sim \mathcal{H}\mathcal{N}(0.0, 1.0), \\ b_{\tau }&\sim \mathcal{H}\mathcal{N}(0.0, 0.2), \\ \beta&\sim \mathcal {B}(25, 25), \\ \sigma _v&\sim \mathcal{H}\mathcal{N}(0.0, 0.1), \\ \sigma _a&\sim \mathcal{H}\mathcal{N}(0.0, 0.1), \\ \sigma _{\tau }&\sim \mathcal{H}\mathcal{N}(0.0, 0.05). \end{aligned}$$

The notation $\mathcal{T}\mathcal{N}(\mu , \sigma , a, b)$ denotes a truncated normal distribution with mean $\mu$, SD $\sigma$, and support restricted to the interval [*a*, *b*]. The notation $\mathcal{H}\mathcal{N}(\mu , \sigma ^2)$ refers to a half-normal distribution – a normal distribution centered at $\mu$ and with SD $\sigma$, truncated to the positive real line. Finally, $\mathcal {B}(\alpha , \beta )$ denotes a beta distribution with shape parameters $\alpha$ and $\beta$ on the interval [0, 1].

Following the standard amortized Bayesian inference (ABI) setting, we generate a data set of simulated data sets, $\smash {\mathcal {D} = \{\theta ^{(b)}_{1:T}, \phi , \eta ^{(b)}, x^{(b)}_{1:T}\}_{b=1}^B}$, and use this to train a neural density estimator $F{\psi }$ that approximates the full posterior $q_{\psi }(\theta _{1:T}, \phi , \eta \mid x_{1:T})$, implemented as a conditional normalizing flow (see Papamakarios et al., 2021). The training objective is to minimize the following loss in expectation over the full non-stationary generative model:

14 $$\begin{aligned} \mathcal {L}(\psi ) = \mathbb {E}_{(\theta _{1:T}, \phi , \eta , x_{1:T}) \sim \mathcal {D}}\left[ -\log q_{\psi }(\theta _{1:T}, \phi , \eta \,|\,x_{1:T}) \right] , \end{aligned}$$

which we approximate using samples from the simulation-based training set $\mathcal {D}$. This online training procedure – based on on-the-fly simulation – follows the approach of Radev et al. (2020) and makes overfitting unlikely.

We used the BayesFlow Python library (Radev et al., 2023) to implement and train the neural approximator. Our architecture comprises a hierarchical summary network, following the architecture used by Elsemüller et al. (2024), along with two dedicated inference networks. The summary network is implemented using three bidirectional long short-term memory (LSTM) layers with 512, 256, and 128 hidden units, respectively. For inference, we employ two invertible neural networks (Radev et al., 2020): one tailored to time-varying parameters and the other to static parameters. The network handling time-varying parameters comprises eight coupling layers that utilize an interleaved design of affine and spline transformations. The static parameter network mirrors this architecture but uses six coupling layers instead. The neural approximator was trained online for 100 epochs, each comprising 1000 iterations with a batch size of 32, resulting in a total of $N =$ 3,200,000 simulated data sets. The initial learning rate was set to 0.0005 and gradually reduced to zero using a cosine decay schedule. All code and data are available in our GitHub repository at https://github.com/LuSchumacher/vicarious-functioning-nsddm.

## Footnotes

1 A term Brunswik adapted from the behaviorist Walter S. Hunter (1932).

2 Representative design departs from the commonly used systematic design (i.e., orthogonal manipulation of variables) by attempting to preserve environmental properties (Dhami et al., 2004).

3 Brunswik distinguishes between different types of cues that can be attributed more to the individual or more to the environment. For example, the size of an object can be conveyed by the change in the retinal projection (individual side) or by the distance to the object (environmental side; Brunswik & Cruikshank, 1937). As this distinction makes no contribution to the description of vicarious mediation and functioning, it is disregarded here. For a detailed description, see Brunswik (1952) or Hammond and Stewart (2001).

4 Note that the ecological validity in Eq. 6 was represented as $|P(a\,|\,\text {Pattern}) - 0.5|$ (cf. rightmost column in Table 1). Therefore, a larger value reflects a higher overall ecological validity of a cue pattern. This simple metric was again used for illustration in Fig. 5.

## Declarations

### Code availability

Analysis code is available at https://github.com/LuSchumacher/vicarious-functioning-nsddm.

### Open practices statements

Data, materials, and analysis code are available (see above).
